# Supplementary figures and images for: Design and antiviral assessment of a panel of fusion proteins targeting human papillomavirus type 16
Source: PLoS One. 2024 Oct 25;19(10):e0311137. doi: 10.1371/journal.pone.0311137 (PMC11508125; doi:10.1371/journal.pone.0311137)

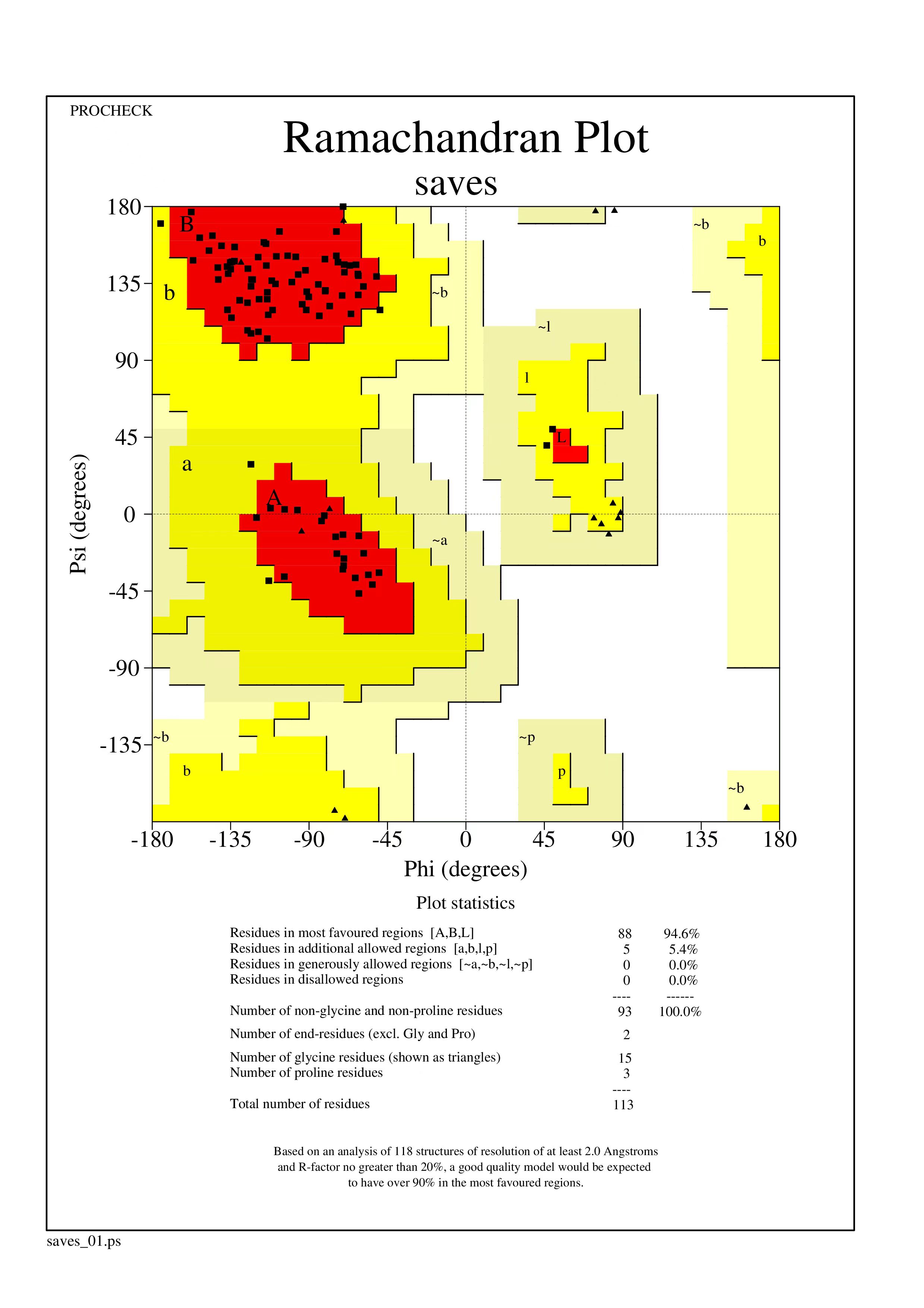

Supplement: S1 Fig — (JPG) [file pone.0311137.s003.jpg]

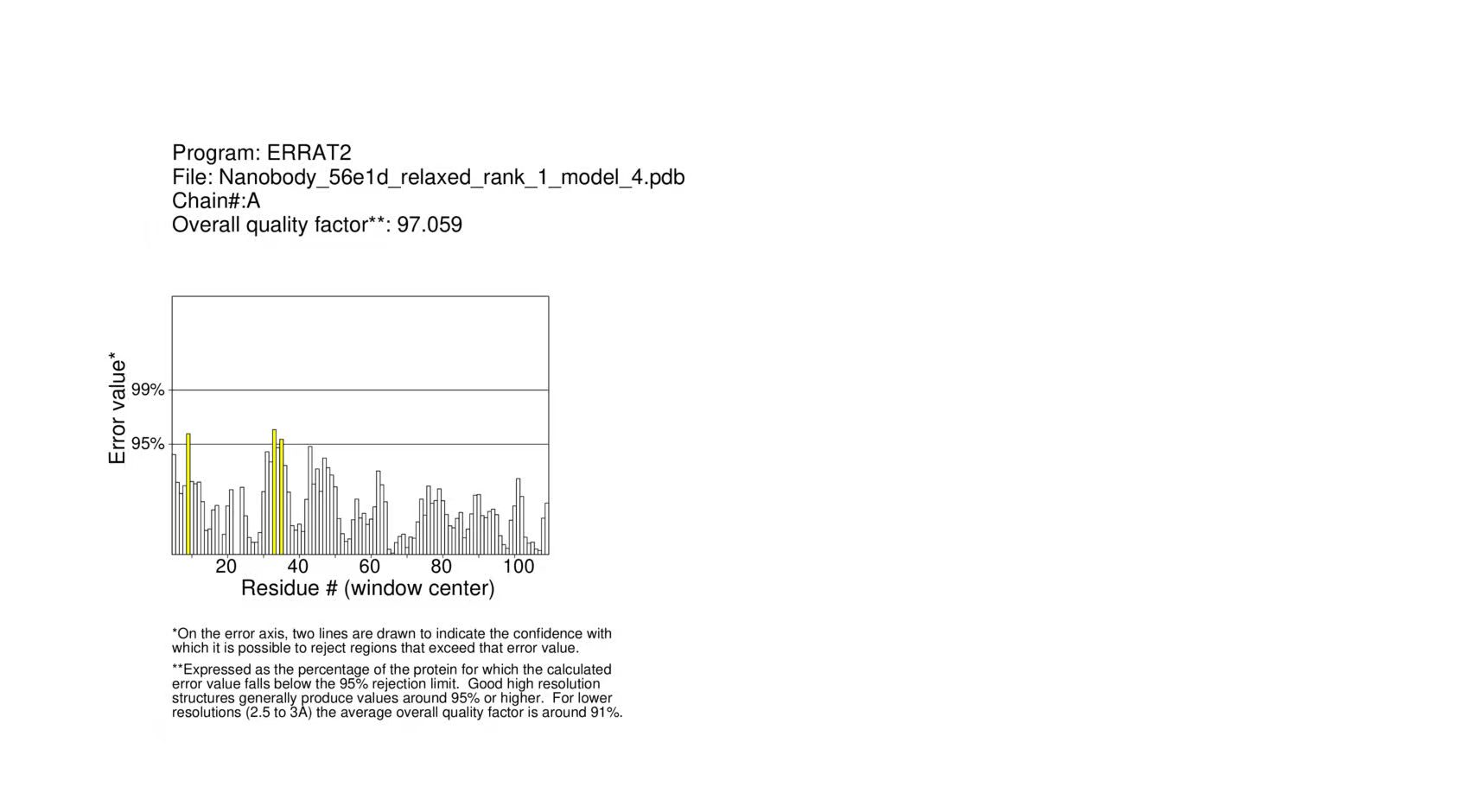

Supplement: S2 Fig — (JPG) [file pone.0311137.s004.jpg]
