# Supplementary material for: Design and antiviral assessment of a panel of fusion proteins targeting human papillomavirus type 16
Source: PLoS One. 2024 Oct 25;19(10):e0311137. doi: 10.1371/journal.pone.0311137 (PMC11508125; doi:10.1371/journal.pone.0311137)
Supplement: Attachment — Submitted filename: PLOS One Review_Round 2.docx [file pone.0311137.s006.docx]

PLOS One Review

Round 2

Line 48 – the term” physical therapy” has a very specific meaning, and their usage in this context is incorrect. The authors should state what clinical intervention for atypical hyperplasia/neoplasia they are referencing, such as Cryotherapy, Laser ablation, Loop electrosurgical excision procedure (LEEP). ...Cone biopsy, etc.
