# Supplementary material for: Design and antiviral assessment of a panel of fusion proteins targeting human papillomavirus type 16
Source: PLoS One. 2024 Oct 25;19(10):e0311137. doi: 10.1371/journal.pone.0311137 (PMC11508125; doi:10.1371/journal.pone.0311137)
Supplement: Attachment — Submitted filename: Response to Reviewers.docx [file pone.0311137.s007.docx]

Response to Reviewers

*1. Line 48 – the term” physical therapy” has a very specific meaning, and their usage in this context is incorrect. The authors should state what clinical intervention for atypical hyperplasia/neoplasia they are referencing, such as Cryotherapy, Laser ablation, Loop electrosurgical excision procedure (LEEP). ...Cone biopsy, etc.*

Response: Thank you for your kind suggestion. We have revised this section. (line 46-49)

*2.* *Please review your reference list to ensure that it is complete and correct. If you have cited papers that have been retracted, please include the rationale for doing so in the manuscript text, or remove these references and replace them with relevant current references. Any changes to the reference list should be mentioned in the rebuttal letter that accompanies your revised manuscript. If you need to cite a retracted article, indicate the article’s retracted status in the References list and also include a citation and full reference for the retraction notice.*

Response: Thank you for your kind reminder. We have revised the reference 19 correctly. (line 382-384)
